# Relationship Between Thoracic CT Severity Score and Scadding Staging With PFT, DLCO, and 6MWT in Patients With Sarcoidosis

**DOI:** 10.1155/carj/1655729

**Published:** 2025-11-19

**Authors:** Muhammet Ali Takeş, Celalettin Korkmaz, Necdet Poyraz, Mücahit Yılmaz, Soner Demirbaş

**Affiliations:** ^1^Department of Pulmonary Diseases, Faculty of Medicine, Necmettin Erbakan University, Konya, Turkey; ^2^Department of Radiology, Faculty of Medicine, Necmettin Erbakan University, Konya, Turkey

**Keywords:** 6-minute walk test, DLCO, pulmonary function test, sarcoidosis, Scadding staging, Thoracic CT Severity Score

## Abstract

**Background:**

Chest computed tomography (CT) is widely used in the diagnosis and follow-up of diffuse parenchymal lung diseases like sarcoidosis. This study investigated the relationship between the Thoracic CT Severity Score (TCTSS) and parameters from pulmonary function tests (PFTs), diffusing capacity for carbon monoxide (DLCO), and the 6-min walk test (6MWT) in sarcoidosis patients, comparing its utility to the radiograph-based Scadding staging system.

**Methods:**

This retrospective study analyzed data from 60 sarcoidosis patients with concurrent chest radiograph, CT, PFT, DLCO, and 6MWT results. Patient demographics, TCTSS, Scadding stage, PFT parameters (forced expiratory volume in one second [FEV1], forced vital capacity [FVC], FEV1/FVC, PEF, and FEF25-75), DLCO, DLCO/VA, and 6MWT parameters (initial SpO2, final SpO2, desaturation percentage, distance, and predicted distance percentage) were collected. Correlations between these functional parameters and both TCTSS and Scadding stages were analyzed to compare the strengths of the two staging systems.

**Results:**

TCTSS showed moderate negative correlations with FEV1% (*r* = −0.44, *p* < 0.001), FVC% (*r* = −0.477, *p* < 0.001), DLCO (*r* = −0.522, *p* < 0.001), final 6MWT SpO2 (*r* = −0.417, *p*=0.001), and 6MWT% (*r* = −0.511, *p* < 0.001). Weaker correlations were observed between TCTSS and initial SpO2, desaturation, and 6MWT distance. The Scadding stage also demonstrated negative correlations with these parameters, although generally weaker for FEV1% and FVC%. TCTSS exhibited stronger negative correlations with FEV1%, FVC%, DLCO, and 6MWT% compared to Scadding staging. Conversely, the Scadding stage correlated more strongly with FVC (liters), FEV1 (liters), FEV1/FVC, DLCO/VA, initial SpO2, final SpO2, and 6MWT distance. Desaturation showed similar low-to-moderate positive correlations with both staging systems.

**Conclusions:**

This study demonstrates that TCTSS significantly correlates with most functional parameters, exhibiting stronger correlations for key measures (FVC%, FEV1%, DLCO, and 6MWT%) compared to the Scadding staging system. TCTSS is a valuable tool that warrants consideration in the follow-up of sarcoidosis patients. Our findings suggest that TCTSS could serve as a potential alternative or complementary system to the Scadding classification, potentially informing the development of a modified combined staging approach. Larger, multicenter studies are necessary to confirm these findings and further explore the role of TCTSS in sarcoidosis management.

## 1. Introduction

Sarcoidosis is a multisystemic granulomatous disease affecting various organs. Intrathoracic involvement, typically presenting as symmetric bilateral hilar lymphadenopathy and/or diffuse pulmonary micronodules, occurs in over 90% of patients [1]. The lungs are the most frequently affected organs (> 90%) [2], and pulmonary involvement is a primary determinant of morbidity and mortality [3]. The estimated annual incidence ranges between 2.3 and 11 per 100,000 individuals [1], with a reported incidence of 4 per 100,000 in Turkey [4].

Sarcoidosis is suspected in some patients when bilateral hilar enlargement is detected incidentally on a chest radiograph taken for other reasons [5]. The characteristic pulmonary involvement pattern is a diffuse interstitial reticulonodular infiltrate, commonly predominant in the upper lobes [6]. While typical radiological findings are present in 60%–70% of patients, computed tomography (CT) offers higher sensitivity than chest radiography for detecting lymphadenopathy and parenchymal abnormalities. High-resolution CT (HRCT) aids in differentiating active disease from irreversible fibrosis [7]. Granulomas can be identified histologically in up to 80% of lung biopsies, even in radiologically unaffected stage I disease [6].

The Thoracic Computed Tomography Severity Score (TCTSS) provides an objective, quantitative method for assessing disease extent by evaluating the degree of involvement in each of the five lung lobes and summing their individual scores [8, 9].

Response to treatment in sarcoidosis is commonly assessed based on a reduction in symptoms, radiological improvement, and improvement in pulmonary function tests (PFTs) [10]. Even patients with a normal posteroanterior chest radiograph may exhibit PFT abnormalities and changes in diffusion capacity. A decrease in 6-min walk test (6MWT) distance is also frequently observed [11].

For evaluating lung involvement in clinical practice, forced vital capacity (FVC), forced expiratory volume in one second (FEV1), and diffusion capacity for carbon monoxide (DLCO) are crucial PFT parameters. FVC is a key parameter for disease monitoring and is often used as a primary endpoint in clinical trials. The FEV1/FVC ratio helps identify patients with airway obstruction [12]. According to a comprehensive review (PubMed, January 2012–December 2022), an FVC < 70% indicates clinically significant pulmonary involvement requiring treatment. Disease progression during follow-up is reflected by developing symptoms or objective loss of lung function, such as a decline in FVC of 5% from baseline or a 10% decrease in DLCO. An improvement exceeding 5% in predicted FVC percentage is considered an effective treatment response [12]. A study comparing mortality rates in patients with FEV1 < 50% versus FEV1 > 80% found a 4.2-fold higher mortality rate in the group with FEV1 < 50% [13]. DLCO reduction in sarcoidosis and other interstitial lung diseases is attributed to a decrease in the alveolocapillary membrane surface area due to parenchymal involvement and fibrosis [14]. Nardi et al. reported decreased DLCO in 90% of 142 Stage 4 sarcoidosis patients [15].

FVC < 70% and DLCO < 60% at diagnosis may signal clinically significant pulmonary pathology warranting treatment [12]. Development of symptoms or objective loss of lung function during follow-up reflects disease progression. Treatment should be considered when FVC decreases by 5% or DLCO by 10% from baseline [16].

The 6MWT is a simple, valuable tool for assessing functional status in sarcoidosis patients, frequently used in initial assessment and as a predictor of survival [12]. Reduced 6MWT distance is associated with increased mortality in sarcoidosis-associated pulmonary hypertension [17].

Since 1961, sarcoidosis has been classified radiologically based on chest radiographs using the Scadding staging system [18]. The clinical relevance of the Scadding system has diminished over time, largely due to technological advancements in imaging. In contemporary practice, CT scanning has become a routine part of the diagnostic workup for sarcoidosis, meaning most patients undergo a scan before a definitive diagnosis is even established. CT scans frequently identify subtle abnormalities, such as affected lymph nodes, minor parenchymal disease, or early fibrosis, which are often missed by conventional chest radiography. It remains unclear how these CT-specific findings relate to the traditional Scadding stages. However, these features may offer a more detailed method to phenotype sarcoidosis patients, highlighting the limitations of current models [19]. To be comprehensive, any new staging approach must therefore incorporate the granular data provided by modern imaging modalities, including CT.

This study aims to investigate the relationships between PFT parameters (FVC, FEV1, FEV1/FVC, PEF, FEF 25–75), diffusion capacity (DLCO and DLCO/VA), and 6MWT parameters (initial peripheral oxygen saturation [SpO2], final SpO2, desaturation percentage, walking distance [meters], and percentage of predicted walking distance) with the Scadding staging system and the TCTSS, and to compare the strengths of these two methods in reflecting functional impairment. To our knowledge, this is the first comprehensive study of this comparison.

## 2. Materials and Methods

### 2.1. Patients and Procedures

This was a retrospective study conducted at the Department of Chest Diseases of our university's Faculty of Medicine Hospital. We included 60 patients over the age of 18 diagnosed with sarcoidosis between January 2015 and May 2024. From a total of 243 sarcoidosis patients followed during this period, inclusion was limited to those with concurrently (within a 3-month window) available chest radiograph, chest CT, PFT, DLCO, and 6MWT data. Patient demographics (age, sex, and comorbidities), TCTSS, Scadding stage, PFT parameters (FVC, FVC%, FEV1, FEV1%, FEV1/FVC, PEF, FEF 25–75, and FEF 25%–75%), diffusion capacity measurements (DLCO and DLCO/VA), and 6MWT parameters (initial SpO2, final SpO2, desaturation percentage, walking distance, and percentage of predicted walking distance (6MWT%)) were extracted from patient records.

Correlations between these functional parameters and TCTSS and Scadding stages were analyzed to compare the strengths and weaknesses of each staging system in reflecting functional status.

### 2.2. Imaging and Scoring

Chest CT scans were performed using a Somatom Drive (Siemens Healthineers, Erlangen, Germany) scanner. Images were reviewed independently by two thoracic radiologists, and scores were determined by consensus. All patients underwent contrast-enhanced CT scans. Scanning parameters included tube voltage 120 kV, tube current-time product 50–100 mAs, slice thickness 3 mm, reconstruction interval 0.6 mm, and matrix 512 × 512. Images were reviewed using standard lung (level: −700 to −550 HU, width: 1000–1500 HU) and mediastinal (level: 30–40 HU, width: 300–350 HU) window settings.

The TCTSS assesses the degree of involvement for each of the five lung lobes. Each lobe is assigned a score according to the degree of involvement: 0: 0% (no involvement), 1: 1%–25% (minimal involvement), 2: 26%–50% (mild involvement), 3: 51%–75% (moderate involvement), and 4: 76%–100% (severe involvement). The “total severity score” was calculated by summing the five lobe scores (possible range, 0–20) [9]. The Scadding classification is defined as Stage 0 (normal PA CXR), Stage 1 (bilateral hilar lymphadenopathy), Stage 2 (bilateral hilar lymphadenopathy and parenchymal infiltration), Stage 3 (parenchymal infiltration without lymphadenopathy), and Stage 4 (parenchymal fibrosis) [18].

### 2.3. Exclusion Criteria

Patients were excluded if they lacked evaluable PFT, DLCO, chest radiograph, or chest CT data; if there were significant time discrepancies (> 3 months) between these tests; or if they had other acute or chronic lung diseases apart from mild asthma that could significantly impact functional parameters.

### 2.4. Statistical Analysis

Data were analyzed using SPSS Version 22.0. Descriptive statistics are reported as frequencies (*n*) and percentages (%) for categorical variables and means ± standard deviations (minimum–maximum) for continuous variables. The chi-square (*χ*^2^) test or Fisher's exact test was used for comparing categorical variables. Normality of continuous variables was assessed using the Kolmogorov–Smirnov and Shapiro–Wilk tests. One-way ANOVA or Welch's test was used to compare continuous variables across multiple groups (Scadding stages and TCTSS stages, where TCTSS stages were based on the lobe scoring system categories: 0%, 1%–25%, 26%–50%, 51%–75%, and 76%–100% applied to the total score range). Post hoc analyses were performed using Tukey's and Tamhane's tests when appropriate. Correlations between continuous variables were assessed using Pearson's correlation analysis. Correlation coefficients were interpreted based on standard guidelines: *r* = 0.05–0.30 (weak), *r* = 0.30–0.40 (low-to-moderate), *r* = 0.40–0.60 (moderate), *r* = 0.60–0.70 (good), *r* = 0.70–0.75 (very good), and *r* = 0.75–1.00 (excellent) [20]. Statistical significance was defined as *p* < 0.05.

### 2.5. Ethical Approval

The study was conducted in accordance with the Declaration of Helsinki and was approved by the Non-Interventional Clinical Research Ethics Committee of Necmettin Erbakan University (approval number: 2024/4981, application ID: 19532, date: May 17, 2024).

## 3. Results

Sixty patients with sarcoidosis were included. Mean age was 52.02 ± 12.84 years (range 18–81), and 71.7% were female. Comorbidities were present in 51.7% of patients, including diabetes mellitus (25%), hypertension (15%), and mild asthma (6%) (Table 1).

The distribution of PFT, diffusion capacity, and 6MWT results for the cohort are presented in Table 2.

According to the Scadding classification, the patient distribution was Stage 0 (21.7%), Stage 1 (31.7%), Stage 2 (31.7%), Stage 3 (6.7%), and Stage 4 (8.3%). The mean total TCTSS for the cohort was 5.32 ± 6.28 (range 0–20) (Table 3).

Analysis of functional parameters across Scadding stages revealed that FVC%, FEV1%, DLCO, DLCO/VA, initial 6MWT SpO2, final 6MWT SpO2, 6MWT distance (meters), and 6MWT% significantly decreased with increasing Scadding stage (*p*=0.001, *p*=0.010, *p* < 0.001, *p*=0.001, *p*=0.013, *p*=0.016, *p*=0.003, and *p*=0.034, respectively). 6MWT desaturation significantly increased with increasing Scadding stage (*p*=0.002) (Table 4).

When PFT and diffusion capacity measurements were analyzed according to TCTSS, FVC%, FEV1%, DLCO, DLCO/VA, final 6MWT SpO2, and 6MWT% significantly decreased as TCTSS increased (*p*=0.012, *p*=0.019, *p* < 0.001, *p*=0.005, *p*=0.005, and *p*=0.005, respectively). No significant differences were observed for FVC (liters), FEV1 (liters), FEV1/FVC, PEF, FEF 25–75, initial 6MWT SpO2, desaturation, or 6MWT distance (meters) across TCTSS stages (*p* > 0.05) (Table 5).

Correlation analysis (Table 6) showed that the Scadding stage had low-to-moderate negative correlations with FVC% (*r* = −0.368; *p*=0.004), FVC (liters) (*r* = −0.385; *p*=0.002), FEV1% (*r* = −0.291; *p*=0.024), and FEV1 (liters) (*r* = −0.340; *p*=0.008). Moderate negative correlations were observed between Scadding stage and DLCO (*r* = −0.493; *p* < 0.001), DLCO/VA (*r* = −0.331; *p*=0.010), initial 6MWT SpO2 (*r* = −0.467; *p* < 0.001), final 6MWT SpO2 (*r* = −0.501; *p* < 0.001), 6MWT distance (meters) (*r* = −0.470; *p* < 0.001), and 6MWT% (*r* = −0.462; *p* < 0.001). A low-to-moderate positive correlation was found between Scadding stage and 6MWT desaturation (*r* = 0.374; *p*=0.003).

Correlation analysis (Table 7) showed that TCTSS had moderate negative correlations with FVC% (*r* = −0.477, *p* < 0.001), FEV1% (*r* = −0.441, *p* < 0.001), DLCO (*r* = −0.522, *p* < 0.001), final 6MWT SpO2 (*r* = −0.417, *p*=0.001), and 6MWT% (*r* = −0.511, *p* < 0.001). A weak negative correlation was found with initial 6MWT SpO2 (*r* = −0.296; *p*=0.022). Low-to-moderate correlations were observed with 6MWT desaturation (positive correlation, *r* = 0.370; *p*=0.004) and 6MWT distance (meters) (negative correlation, *r* = −0.341; *p*=0.008). No significant correlations were found between TCTSS and FVC (liters), FEV1 (liters), FEV1/FVC, PEF, FEF 25%–75%, FEF 25–75 (L/s), or DLCO/VA (*p* > 0.05).

Comparing the correlation strengths (absolute values of *r*) between the two staging systems and clinical parameters (PFT, diffusion capacity, and 6MWT data), FVC%, FEV1%, DLCO, and 6MWT% showed stronger negative correlations with TCTSS. However, FVC (liters), FEV1 (liters), FEV1/FVC, DLCO/VA, initial SpO2, final SpO2, and 6MWT distance (meters) showed stronger correlations with the Scadding stage. 6MWT desaturation showed similar low-to-moderate positive correlations with both staging systems (*r* = 0.374 for Scadding, *r* = 0.370 for TCTSS).

## 4. Discussion

Chest radiography remains a common initial radiological technique for assessing pulmonary involvement in sarcoidosis, and the Scadding staging system, developed in 1961 based on chest radiographs, has been used for radiological classification and outcome assessment [18]. Despite its long-term use, the utility of the Scadding system is increasingly challenged by advances in imaging technology, particularly CT [19]. CT provides a more detailed assessment of parenchymal disease and lymphadenopathy, often revealing abnormalities not visible on chest radiographs.

Previous studies have consistently shown an association between increasing Scadding stage and decreased lung function, particularly FVC [21–23]. PFTs are reportedly impaired in approximately 20% of Stage 1 patients and 40%–80% of patients with parenchymal involvement [24]. Several previous studies have associated Stage 1 disease with mild PFT abnormalities, better than those in Stages 2 and 3, while Stage 4 patients have the worst pulmonary function, with 75% succumbing to respiratory complications, including pulmonary hypertension and chronic respiratory failure [15, 25–27]. Our findings support these observations, demonstrating statistically significant low-to-moderate negative correlations between Scadding stage and FVC% (*r* = −0.368), FVC (liters) (*r* = −0.385), FEV1% (*r* = −0.291), FEV1 (liters) (*r* = −0.340), DLCO (*r* = −0.493), and DLCO/VA (*r* = −0.331). These findings are consistent with previous reports from Turkey and elsewhere, which also found significant negative correlations between radiographic stage and FVC and DLCO [28–30], and FEV1% and FVC% [29]. Our observation of significant decreases in FVC%, FEV1%, DLCO, and DLCO/VA across Scadding stages aligns with the literature [23, 29, 30]. The data presented in Table 4, particularly for Stage 3 patients, show some values that deviate from the general trend of declining function with increasing stage. This is likely attributable to the small sample size in this stage (*n* = 4), reducing the robustness of the mean values.

Thoracic CT is recognized as a more accurate method for identifying mediastinal lymphadenopathy and subtle pulmonary parenchymal changes and is widely used for initial evaluation and follow-up in sarcoidosis [26]. Studies have indicated that HRCT findings correlate better with the severity of PFT changes than PA CXR stages [31]. Prior research has explored correlations between specific CT patterns and PFTs, showing negative correlations between CT scores for consolidation, ground-glass opacities, reticular patterns, and air trapping with various functional parameters like FVC, FEV1, FEV1/FVC, DLCO, RV, and TLC [31–35]. However, a universally accepted CT-based staging system for sarcoidosis equivalent to Scadding staging has not yet been established.

Our study specifically evaluated the TCTSS, a quantitative measure of overall parenchymal involvement extent, in relation to functional parameters. We found moderate negative correlations between TCTSS and FVC% (*r* = −0.477), FEV1% (*r* = −0.441), and DLCO (*r* = −0.522). These results suggest that TCTSS effectively captures the severity of parenchymal disease that impacts lung volumes and gas exchange.

Our findings suggest that TCTSS may be more sensitive to changes in overall lung volumes, expiratory flow rates, gas exchange capacity, and exercise performance, as it provides a quantitative measure of the extent of parenchymal disease. The stronger correlations with FEV1% and FVC% using TCTSS compared to Scadding align with the hypothesis that a quantitative CT score is better correlated with PFT severity than traditional radiographic stages, which are less sensitive to subtle parenchymal disease. The stronger correlation with FEV1% and FVC% using TCTSS compared to Scadding aligns with the hypothesis that a quantitative CT score is better correlated with PFT severity than traditional radiographic stages, which are less sensitive to subtle parenchymal disease.

Conversely, the Scadding stage showed stronger correlations with FVC (liters), FEV1 (liters), FEV1/FVC, DLCO/VA, initial SpO2, final SpO2, and 6MWT distance (meters). This might be explained by the nature of the Scadding system, which includes lymph node involvement (Stages 1 and 2) and fibrosis (Stage 4) as distinct categories, potentially capturing a broader spectrum of disease impact beyond just the extent of parenchymal disease as quantified by TCTSS. For example, lymphadenopathy in early stages might affect airway caliber or pulmonary blood flow independent of widespread parenchymal infiltration, influencing parameters like FEV1/FVC or DLCO/VA differently than parenchymal extent alone. Furthermore, the Scadding classification's clear distinction of the fibrotic stage (Stage 4) correlates strongly with severe functional deficits, including significant desaturation and reduced absolute 6MWT distance, which may not be solely captured by the TCTSS score if it focuses primarily on active inflammation/granuloma extent rather than fibrotic burden. The significant desaturation observed in our study, correlating positively with Scadding stage (*r* = 0.374), especially in Stage 4 patients, supports the notion that advancing radiographic stage, particularly with fibrosis, is linked to impaired gas exchange during exertion.

Exercise capacity, as assessed by the 6MWT, is a crucial prognostic indicator in sarcoidosis [11, 12]. Our mean 6MWT distance of 440.45 ± 118.34 m is comparable to other sarcoidosis cohorts [36–39]. Consistent with previous studies [12, 36], we found significant decreases in 6MWT distance and 6MWT% with increasing Scadding stage (Table 4), with Stage 4 patients walking significantly shorter distances than Stages 0 and 1 patients. We also observed a significant negative correlation between Scadding stage and 6MWT distance (*r* = −0.470) and 6MWT% (*r* = −0.462) and a significant positive correlation with desaturation (*r* = 0.374). While TCTSS also showed negative correlations with 6MWT distance (*r* = −0.341) and 6MWT% (*r* = −0.511) and a positive correlation with desaturation (*r* = 0.370), the absolute distance and desaturation correlations were stronger for the Scadding stage. This might again relate to Scadding stages, particularly Stages 1 and 4, capturing aspects of disease burden (lymphadenopathy and fibrosis) that significantly impair exercise capacity and oxygenation response beyond the mere extent of parenchymal involvement quantified by TCTSS. The finding that Scadding Stage 1 patients walked significantly further than Stage 2 patients, despite both potentially having parenchymal disease by CT (as seen in the TCTSS distribution across Scadding stages in Table 4), highlights the potential divergence between the systems and suggests Scadding Stage 1 (lymph nodes only by CXR) represents a less functionally impaired group on average than Scadding Stage 2 (lymph nodes + parenchyma by CXR), which is also reflected in the TCTSS distribution where Scadding Stage 2 has a higher proportion of patients with significant TCTSS stages (Table 4: Stage 2 has patients in TCTSS Stages 1, 2, 3, and 4, whereas Stage 1 is almost entirely TCTSS Stage 1).

In summary, TCTSS correlates well with core functional parameters reflecting overall lung volume and gas exchange capacity (FVC%, FEV1%, DLCO, 6MWT%). The Scadding stage, while also correlating significantly with many parameters, shows stronger associations with parameters reflecting specific manifestations or overall disease burden across distinct stages (FVC, FEV1 liters, FEV1/FVC, DLCO/VA, SpO2, 6MWT distance). This suggests that TCTSS is a good measure of parenchymal disease severity influencing global function, whereas Scadding might reflect disease characteristics and progression more broadly, including extraparenchymal components and fibrotic consequences.

Our study demonstrates that TCTSS provides valuable quantitative information related to pulmonary function that complements or, for certain parameters, surpasses the traditional Scadding staging system. This supports the idea that CT-based quantitative scoring is relevant for assessing disease severity and functional impact. The differing strengths of correlation suggest that neither system is universally superior for all functional parameters and highlight the need for a system that incorporates the detailed information provided by CT.

### 4.1. Limitations of Study

The major limitations of our study include its retrospective design, which restricts causal inference and introduces potential selection bias. Furthermore, the requirement for concurrent CT, PFT, DLCO, and 6MWT data limited the sample size to 60 patients out of 243 eligible patients, potentially impacting the statistical power and generalizability of the findings. This relatively small sample size, particularly the low numbers in Scadding Stages 3 and 4 (4 and 5 patients, respectively) and higher TCTSS stages (Table 3), may have influenced the statistical comparisons across stages and correlation strengths for some parameters. Being a single-center study also limits the generalizability of the results. The study lacked longitudinal data, which would be beneficial for assessing the predictive value of TCTSS and the Scadding stage for functional decline or treatment response over time.

## 5. Conclusion

This study is, to our knowledge, the first comprehensive comparison of the TCTSS and the Scadding staging system in relation to a wide range of PFT, DLCO, and 6MWT parameters in sarcoidosis patients. We found that TCTSS significantly correlated with most functional parameters, showing stronger negative correlations with FVC%, FEV1%, DLCO, and 6MWT% compared to the radiograph-based Scadding stage. Conversely, the Scadding stage correlated more strongly with absolute FVC and FEV1, FEV1/FVC, DLCO/VA, SpO2 measures, and 6MWT distance.

Our findings demonstrate that TCTSS is a valuable quantitative measure of radiological pulmonary involvement that correlates significantly with functional status in sarcoidosis. Given its ability to quantify parenchymal severity, TCTSS provides complementary information to the Scadding system and may be particularly useful in assessing parameters directly impacted by the extent of parenchymal disease. These results suggest TCTSS warrants consideration as a valuable tool in the follow-up of patients with sarcoidosis, potentially as an alternative or a complementary tool to the Scadding system. The differing strengths of correlation observed highlight the potential benefit of integrating quantitative CT assessment into a more comprehensive staging approach. Based on our findings, we propose that future staging systems could consider subdividing radiographic stages where parenchymal involvement is present (e.g., Scadding Stages 2, 3, and 4) based on quantitative CT severity, potentially into substages (e.g., Stages 2a, 2b, 2c, 2d, 3a, 3b, 3c, 3d, 4a, 4b, 4c, 4d) reflecting increasing radiological severity by CT. Larger, multicenter, prospective studies are essential to validate these findings and further explore the optimal role of TCTSS, alone or in combination with other measures, in the comprehensive assessment and management of sarcoidosis.

## Figures and Tables

**Table 1:** Demographic and comorbidity data of patients.

Characteristic	All patients *n* = 60 (%)
Age (years)	52.02 ± 12.84 (18–81)
Sex	
Female	43 (71.7)
Male	17 (28.3)
Comorbidity	31 (51.7)
Diabetes mellitus (DM)	15 (25.0)
Hypertension (HT)	9 (15.0)
Asthma	4 (6.0)
Other	3 (5.0)

*Note: n*: number, (%): percentage.

Abbreviations: DM, diabetes mellitus; HT, hypertension.

**Table 2:** Distribution of PFT, diffusion capacity, and 6MWT data in patients.

PFT and 6MWT parameters	All patients *n* = 60 mean ± SD (min–max)
FVC (%)	81.53 ± 18.78 (27–124)
FVC (L)	2.81 ± 1.08 (1–6)
FEV1 (%)	83.77 ± 18.79 (31–123)
FEV1 (L)	2.30 ± 0.86 (1–5)
FEV1/FVC (%)	82.30 ± 7.00 (65–100)
PEF (L/s)	5.73 ± 2.13 (2.33–11.57)
FEF 25–75 (%)	99.00 ± 35.32 (30–177)
FEF 25–75 (L/s)	2.57 ± 1.08 (1–6)
DLCO (mL/min/mmHg)	92.45 ± 21.62 (36–149)
DLCO/VA (mL/min/mmHg/L)	88.27 ± 19.28 (36–136)
6MWT initial SpO2	95.83 ± 1.64 (91–99)
6MWT final SpO2	91.77 ± 5.50 (70–98)
6MWT desaturation (%)	4.13 ± 4.73 (0–23)
6MWT distance (m)	440.45 ± 118.34 (154–755)
6MWT (%)	77.21 ± 16.25 (31–107)

*Note:* Mean ± SD (min–max): mean ± standard deviation (minimum–maximum). FEV1: forced expiratory volume in one second, FEF 25–75: maximal mid-expiratory flow rate, DLCO: carbon monoxide diffusing capacity, PEF: peak expiratory volume, DLCO/VA: DLCO/alveolar ventilation ratio.

Abbreviation: FVC, forced vital capacity.

**Table 3:** Distribution of Scadding stages and thoracic CT severity scores in patients.

	All patients *n* = 60 (%)
Scadding stage	
Stage 0	13 (21.7)
Stage 1	19 (31.7)
Stage 2	19 (31.7)
Stage 3	4 (6.7)
Stage 4	5 (8.3)
Right upper lobe	
No involvement	27 (45.0)
0%–25%	13 (21.7)
25%–50%	9 (15.0)
50%–75%	6 (10.0)
75%–100%	5 (8.3)
Middle lobe	
No involvement	29 (48.3)
0%–25%	15 (25.0)
25%–50%	8 (13.3)
50%–75%	3 (5.0)
75%–100%	5 (8.3)
Right lower lobe	
No involvement	29 (48.3)
0%–25%	14 (23.3)
25%–50%	7 (11.7)
50%–75%	5 (8.3)
75%–100%	5 (8.3)
Left upper lobe	
No involvement	29 (48.3)
0%–25%	12 (20.0)
25%–50%	9 (15.0)
50%–75%	5 (8.3)
75%–100%	5 (8.3)
Left lower lobe	
No involvement	29 (48.3)
0%–25%	14 (23.3)
25%–50%	8 (13.3)
50%–75%	4 (6.7)
75%–100%	5 (8.3)
Total score	5.32 ± 6.28 (0–20)

*Note: n*: number, (%): percentage.

**Table 4:** Distribution of PFT, DLCO, 6MWT, and thoracic CT severity scores data according to Scadding stages in patients.

Parameter	Scadding Stage 0 (*n* = 13) Mean ± SD	Scadding Stage 1 (*n* = 19) Mean ± SD	Scadding Stage 2 (*n* = 19) Mean ± SD	Scadding Stage 3 (*n* = 4) Mean ± SD	Scadding Stage 4 (*n* = 5) Mean ± SD	*p* value	Post hoc^∗∗∗^
FVC (%)	91.08 ± 13.15	87.58 ± 16.46	74.42 ± 17.37	86.50 ± 4.20	56.80 ± 24.38	0.001^∗^	a-e: 0.002; b-e: 0.004
FVC (L)	3.22 ± 0.83	3.09 ± 1.18	2.47 ± 0.98	2.98 ± 1.02	1.77 ± 0.97	0.077^∗∗^	
FEV1 (%)	90.31 ± 13.93	89.47 ± 16.51	78.05 ± 19.22	90.25 ± 8.30	61.60 ± 24.07	0.010^∗^	a-e: 0.021; b-e: 0.018
FEV1 (L)	2.56 ± 0.64	2.54 ± 0.90	2.07 ± 0.87	2.47 ± 0.78	1.49 ± 0.75	0.067^∗^	
FEV1/FVC (%)	79.15 ± 6.89	82.58 ± 7.11	83.11 ± 7.62	82.75 ± 4.27	86.00 ± 5.14	0.374^∗^	
PEF (L/s)	5.45 ± 1.36	6.54 ± 2.20	5.42 ± 2.47	5.45 ± 1.79	4.93 ± 2.26	0.415^∗^	
FEF 25–75 (%)	95.23 ± 36.55	101.16 ± 40.00	97.95 ± 36.31	101.50 ± 26.14	102.60 ± 25.56	0.990^∗^	
FEF 25–75 (L/s)	2.63 ± 0.81	2.73 ± 1.01	2.48 ± 1.39	2.63 ± 0.81	2.15 ± 0.54	0.859^∗^	
DLCO (mL/min/mmHg)	99.54 ± 12.61	106.05 ± 17.53	84.42 ± 20.11	83.75 ± 15.69	59.80 ± 16.63	< 0.001^∗^	a-e: 0.001; b-c: 0.003; b-e < 0.001
DLCO/VA (mL/min/mmHg/L)	88.69 ± 14.13	101.42 ± 16.81	82.47 ± 15.89	75.50 ± 18.35	69.40 ± 25.14	0.001^∗^	b-c: 0.009; b-e: 0.003
6MWT initial SpO2	96.69 ± 1.75	96.26 ± 1.19	95.26 ± 1.79	95.75 ± 0.50	94.20 ± 1.30	0.013^∗^	a-e: 0.024
6MWT final SpO2	94.54 ± 2.43	93.89 ± 2.37	90.11 ± 5.09	93.75 ± 1.89	81.20 ± 9.03	0.016^∗∗^	a-c: 0.028
6MWT desaturation (%)	2.15 ± 1.40	2.58 ± 1.98	5.16 ± 4.89	2.00 ± 2.16	13.00 ± 7.96	0.002^∗^	a-e < 0.001; b-e < 0.001; c-e: 0.001; d-e: 0.001
6MWT distance (m)	480.00 ± 65.58	496.95 ± 139.05	396.95 ± 88.96	410.50 ± 120.13	312.20 ± 97.66	0.003^∗^	a-e: 0.032; b-c: 0.042; b-e: 0.009
6MWT (%)	87.00 ± 11.48	81.57 ± 13.02	72.47 ± 12.89	76.50 ± 13.42	53.80 ± 25.70	0.034^∗∗^	a-c: 0.024
Total TCT Severity Score	1.92 ± 5.60	0.95 ± 1.87	8.58 ± 4.47	10.00 ± 7.70	14.60 ± 5.50	< 0.001^∗∗^	a-c: 0.017; a-e: 0.029; b-c < 0.001; b-e: 0.045
TCT Severity Score Stage; *n* (%)							
Stage 1 (minimal)	12 (92.3)	18 (94.7)	6 (31.6)	2 (50.0)	0		
Stage 2 (mild)	0	1 (5.3)	6 (31.6)	0			
Stage 3 (moderate)	0	0	6 (31.6)	1 (25.0)	2 (40.0)		
Stage 4 (severe)	1 (7.7)	0	1 (5.3)	1 (25.0)	2 (40.0)		

*Note:* Mean ± SD: mean ± standard deviation. FEV1: forced expiratory volume in one second, PEF: peak expiratory volume, FEF 25–75: maximal mid-expiratory flow rate, DLCO: carbon monoxide diffusion capacity, DLCO/VA: DLCO/alveolar ventilation ratio.

Abbreviations: 6MWT, 6-minute walk test; FVC, forced vital capacity.

^∗^One-way ANOVA.

^∗∗^Welch test.

^∗∗∗^Post hoc test (Tukey or Tamhane).

**Table 5:** Distribution of PFT, DLCO, and 6MWT data according to thoracic CT severity score stages in patients.

Parameter	CT score Stage 1 (*n* = 38) Mean ± SD	CT score Stage 2 (*n* = 8) Mean ± SD	CT score Stage 3 (*n* = 9) Mean ± SD	CT score Stage 4 (*n* = 5) Mean ± SD	*p* value	Post hoc^∗∗∗^
FVC (%)	86.16 ± 17.63	80.38 ± 14.86	75.56 ± 10.95	59.00 ± 27.94	0.012^∗^	a-d: 0.010
FVC (L)	2.96 ± 1.05	2.59 ± 1.07	2.30 ± 0.71	2.81 ± 1.08	0.398^∗^	
FEV1 (%)	87.32 ± 17.87	86.00 ± 17.71	79.67 ± 12.96	60.60 ± 23.20	0.019^∗^	a-d: 0.013
FEV1 (L)	2.40 ± 0.83	2.20 ± 0.93	1.94 ± 0.63	2.35 ± 1.30	0.528^∗^	
FEV1/FVC (%)	81.21 ± 6.81	84.50 ± 9.39	83.56 ± 5.59	84.80 ± 8.67	0.461^∗^	
PEF (L/s)	5.87 ± 2.00	5.79 ± 3.06	4.78 ± 1.68	6.31 ± 2.52	0.517^∗^	
FEF 25–75 (%)	97.76 ± 37.80	108.13 ± 40.66	100.56 ± 29.63	91.00 ± 16.53	0.844^∗^	
FEF 25–75 (L/s)	2.55 ± 1.08	2.64 ± 1.58	2.32 ± 0.83	2.57 ± 1.08	0.667^∗^	
DLCO (mL/min/mmHg)	101.39 ± 17.07	74.25 ± 23.06	80.56 ± 17.58	75.00 ± 23.06	< 0.001^∗^	a-b: 0.002; a-c: 0.018; a-d: 0.020
DLCO/VA (mL/min/mmHg/L)	93.95 ± 17.13	72.25 ± 20.49	77.00 ± 18.68	91.00 ± 14.88	0.005^∗^	a-b: 0.013
6MWT initial SpO2	96.16 ± 1.66	95.63 ± 1.59	94.89 ± 1.26	95.40 ± 1.81	0.179^∗^	
6MWT final SpO2	93.47 ± 2.88	90.63 ± 6.67	86.78 ± 7.94	89.60 ± 8.62	0.005^∗^	a-c: 0.004
6MWT desaturation (%)	2.79 ± 2.05	5.00 ± 7.03	8.11 ± 6.86	5.80 ± 7.25	0.180^∗∗^	
6MWT distance (m)	468.00 ± 125.62	406.88 ± 76.97	382.22 ± 83.09	389.60 ± 126.91	0.124^∗∗^	
6MWT (%)	82.10 ± 13.36	73.00 ± 11.75	70.55 ± 21.56	58.80 ± 16.96	0.005^∗^	a-d: 0.009

*Note:* Mean ± SD: mean ± standard deviation. FEV1: forced expiratory volume in one second, PEF: peak expiratory volume, FEF 25–75: maximal mid-expiratory flow rate, DLCO: carbon monoxide diffusion capacity, DLCO/VA: DLCO/alveolar ventilation ratio.

Abbreviations: 6MWT, 6-minute walk test; FVC, forced vital capacity.

^∗^One-way ANOVA.

^∗∗^Welch test.

^∗∗∗^Post hoc test (Tukey or Tamhane).

**Table 6:** Correlation analysis between Scadding stages and PFT, DLCO, and 6MWT data.

PFT, DLCO, 6MWT parameter	Scadding stage		
	*r* value	95% CI	*p* value^∗^
FVC (%)	−0.368	−0.632–−0.259	0.004
FVC (L)	−0.385	−0.552–−0.128	0.002
FEV1 (%)	−0.291	−0.559–−0.123	0.024
FEV1 (L)	−0.340	−0.518–−0.085	0.008
FEV1/FVC (%)	0.263	0.046–0.480	0.042
PEF (L/s)	−0.091	−0.321–0.126	0.492
FEF25-75 (%)	0.053	−0.192–0.254	0.689
FEF25-75 (L/s)	−0.099	−0.302–0.098	0.450
DLCO (mL/min/mmHg)	−0.493	−0.692–−0.367	< 0.001
DLCO/VA (mL/min/mmHg/L)	−0.331	−0.578–−0.127	0.010
6MWT initial SpO2	−0.467	−0.621–−0.191	< 0.001
6MWT final SpO2	−0.501	−0.725–−0.339	< 0.001
6MWT desaturation (%)	0.374	0.255–0.695	0.003
6MWT distance (m)	−0.470	−0.603–−0.229	< 0.001
6MWT (%)	−0.462	−0.704–−0.278	< 0.001

*Note:* FEV1: forced expiratory volume in one second, PEF: peak expiratory volume, FEF 25–75: maximal mid-expiratory flow rate, DLCO: carbon monoxide diffusion capacity, DLCO/VA: DLCO/alveolar ventilation ratio.

Abbreviations: 6MWT, 6-minute walk test; FVC, forced vital capacity.

^∗^Pearson's correlation test.

**Table 7:** Correlation analysis between total thoracic CT severity score and PFT, DLCO, and 6MWT data.

PFT, DLCO, 6MWT parameter	Total thoracic CT severity score		
	*r* value	95% CI	*p* value^∗^
FEV1 (%)	−0.441	−0.637–−0.199	< 0.001
FEV1 (L)	−0.194	−0.473–0.103	0.138
FVC (%)	−0.477	−0.676–−0.230	< 0.001
FVC (L)	−0.210	−0.494–0.106	0.108
FEV1/FVC (%)	0.156	−0.107–0.400	0.233
PEF (L/s)	−0.109	−0.356–0.178	0.413
FEF 25–75 (%)	−0.078	−0.286–0.120	0.553
FEF 25–75 (L/s)	−0.019	−0.227–0.211	0.886
DLCO (mL/min/mmHg)	−0.522	−0.669–−0.338	< 0.001
DLCO/VA (mL/min/mmHg/L)	−0.242	−0.431–−0.026	0.062
6MWT initial SpO2	−0.296	−0.535–−0.071	0.022
6MWT final SpO2	−0.417	−0.646–−0.176	0.001
6MWT desaturation (%)	0.370	0.111–0.614	0.004
6MWT distance (m)	−0.341	−0.547–−0.106	0.008
6MWT (%)	−0.511	−0.689–−0.285	< 0.001

*Note:* FEV1: forced expiratory volume in one second, PEF: peak expiratory volume, FEF 25–75: maximal mid-expiratory flow rate, DLCO: carbon monoxide diffusion capacity, DLCO/VA: DLCO/alveolar ventilation ratio.

Abbreviations: 6MWT: 6-minute walk test; FVC, forced vital capacity.

^∗^Pearson's correlation test.

## Data Availability

The data that support the findings of this study are available from Necmettin Erbakan üniversitesi Tıp Fakültesi. Restrictions apply to the availability of these data, which were used under license for this study. Data are available from the author(s) with the permission of Necmettin Erbakan üniversitesi Tıp Fakültesi.
